# Electrostatic Assembly of Platinum Nanoparticles along Electrospun Polymeric Nanofibers for High Performance Electrochemical Sensors

**DOI:** 10.3390/nano7090236

**Published:** 2017-08-24

**Authors:** Peng Li, Mingfa Zhang, Xueying Liu, Zhiqiang Su, Gang Wei

**Affiliations:** 1 Beijing Key Laboratory of Advanced Functional Polymer Composites, Beijing University of Chemical Technology, Beijing 100029, China; lipeng@mail.buct.edu.cn; 2 State Key Laboratory of Chemical Resource Engineering, Beijing University of Chemical Technology, Beijing 100029, China; zmingfa@outlook.com (M.Z.); liuxueying0426@hotmail.com (X.L.); 3 Faculty of Production Engineering, University of Bremen, D-28359 Bremen, Germany

**Keywords:** electrostatic assembly, polymeric nanofibers, electrospinning, PtNPs, electrochemical sensor

## Abstract

A novel polyacrylonitrile (PAN) nanofibrous membrane conjugated with platinum nanoparticles (PtNPs) was fabricated by electrospinning and electrostatic assembly techniques. In this procedure, PAN was electrospun with 3-aminopropyltriethoxysilane (APS) together as precursor materials. First, amine groups were introduced onto PAN nanofibers, and then the as-prepared negative-charged platinum nanoparticles (PtNPs) were conjugated onto the surface of the amino-modified PAN nanofibers uniformly by the electrostatic interaction-mediated assembly. The fabricated PAN–PtNPs hybrid nanofibrous membrane was further utilized to modify the glassy carbon electrodes and was used for the fabrication of a non-enzymatic amperometric sensor to detect hydrogen peroxide (H_2_O_2_). The electrochemical results indicated that, due to the uniform dispersion of PtNPs and the electrostatic interaction between amine groups and PtNPs, the fabricated PAN–PtNPs nanofibrous membrane-based electrochemical sensor showed excellent electrocatalytic activity toward H_2_O_2_, and the chronoamperometry measurements illustrated that the fabricated sensor had a high sensitivity for detecting H_2_O_2_. It is anticipated that the strategies used in this work will not only guide the design and fabrication of functional polymeric nanofiber-based biomaterials and nanodevices, but also extend their potential applications in energy storage, cytology, and tissue engineering.

## 1. Introduction

Recently, non-enzymatic electrochemical hydrogen peroxide (H_2_O_2_) sensors based on functional hybrid nanomaterials have attracted more and more attention compared to enzymatic sensors because of their advantages such as higher selectivity and sensitivity, simpler fabrication strategy, and lower price [1,2,3]. They possess wide applications in qualitative or quantitative clinical analysis and food safety evaluation [4,5,6]. Compared to other detection techniques such as spectrophotometry [7], chemiluminescence [8], and electrochemical enzymatic sensing [9,10], the non-enzymatic electrochemical H_2_O_2_ sensors show superiority in terms of their simplicity and low cost [11,12]. In particular, although the enzymatic sensors usually have unique selectivity towards a lot of biological analytes, they still possess obvious shortcomings such as susceptibility and a short service life. Previous studies have indicated that non-enzymatic electrochemical sensors could prohibit these disadvantages on one hand, and on the other hand they can obtain higher stability and better reproducibility [13,14,15].

Electrospinning is a simple and efficient technique that is driven by electrical forces to fabricate pure or hybrid polymer nanofibers (PMNFs). This occurs when the electrical forces at the surface of a polymer solution or melt overcome the surface tension and cause an electrically charged jet to be ejected [16,17,18,19]. By this way, we can obtain various PMNFs with adjustable length, diameter, chemical functionality, and mechanical properties by selecting various polymer precursors and changing the electrospinning parameters [19]. For example, by incorporating nanoscale building blocks (NBBs) such as metallic nanoparticles (MNPs) or quantum dots within the spinning dope or onto the surface of PMNFs after spinning, it is possible to adjust the physical and chemical properties of electrospun PMNFs by introducing functional additives with special optical and electrical properties to create novel hybrid nanofibrous materials [20]. In addition, the nanofibrous membrane fabricated by electrospinning has a uniform three-dimensional (3D) porous nanostructure, which consists of thousands of nanofibers with or without particular arrangement. The large surface area and high porosity of the fabricated nanofibrous PMNF membrane could provide a lot of active sites for the immobilization of analytes, affording an enhanced current response for the test molecules/biomolecules [21]. Previously, we prepared a graphene-PMNF hybrid nanofibrous membrane by electrospinning and further utilized the created membrane materials for the fabrication of a high performance electrochemical H_2_O_2_ sensor [22]. The obtained results indicated that it is possible to improve the electrochemical detection of analytes by incoprating other electroactive nanomaterials into the electrospun PMNFs.

Electrode materials are the bridges connecting the sensor system and current conduction system. In the biological detection aspect, oxidation-reduction reactions take place between electrode materials and biological detected objects under a given voltage, and therefore a good electrode material should possess the abilities of precise selectivity, rapid responses, low detection limit, and good electrical conductivity [21,23,24]. Generally speaking, to guarantee both sensing and conducting properties of an electrode material, a catalyst with high selectivity and conductivity is necessary. Previously, MNPs such as Au, Ag, Pt, and Cu NPs have been thought to be potential candidates to fabricate various electrode materials for electrochemical H_2_O_2_ biosensors or sensors due to their good electrochemical activity toward analytes as well as their good electrical conductivity [25,26,27]. Previous studies have indicated that the combination of NBBs with PMNFs to create hybrid nanofibrous membranes is a potential way to fabricate functional electrode materials. We have demonstrated that both one-dimensional (1D) materials like carbon nanotubes [28,29] and two-dimensional (2D) materials like graphene [22] could assist in the better dispersion of MNPs and further improve the electrical conductivity of electrospun PMNFs.

**Figure 1:**
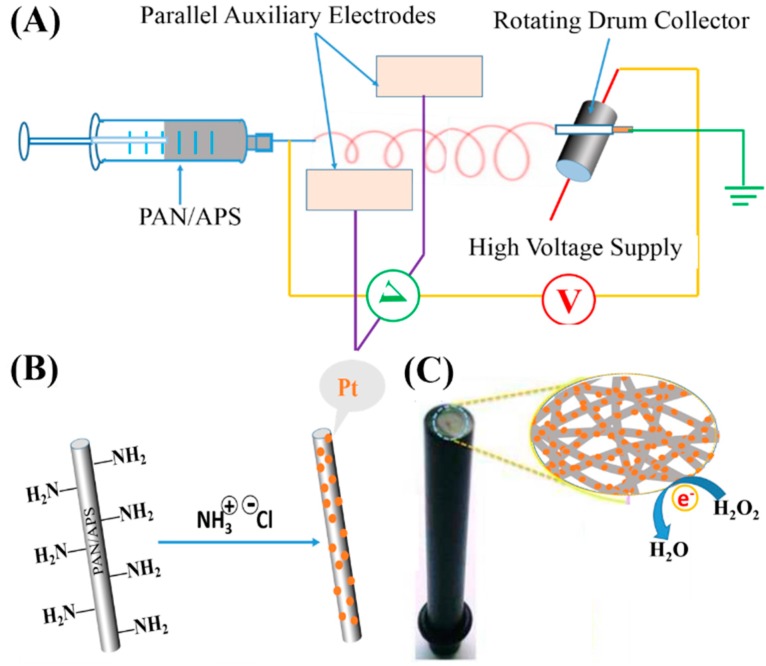
(**A**) Model of a home-made electrospinning instrument for the synthesis of polyacrylonitrile/3-aminopropyltriethoxysilane (PAN/APS) hybrid nanofibers; (**B**) Electrostatic assembly mechanism of platinum nanoparticles (PtNPs) along amino-modified PAN nanofibers; (**C**) PAN–PtNPs hybrid nanofibrous membrane-modified electrode and the potential sensing mechanism of H_2_O_2_.

However, with the pre-processing method, the dispersion of NBBs in electrospun PMNFs is always a challenge. The huge gap in surface energy between NPs and the polymeric matrix may lead to severe aggregation and further affect the final sensing performance [30], and the NBBs that remain trapped inside PMNFs possess a limited role for analytical detection [31,32]. To solve this problem, we first fabricated amino-functionalized polyacrylonitrile (PAN) nanofibers by electrospinning a PAN and 3-aminopropyltriethoxysilane (APS) mixed precursor solution, as shown in Figure 1A. Therefore, the as-synthesized negative-charged platinum nanoparticles (PtNPs) could be conjugated onto the positive-charged PAN/APS nanofibers through the electrostatic interaction (Figure 1B). To fabricate an electrochemical sensor, the created PAN–PtNPs hybrid nanofibrous membrane was further utilized to modify a glassy carbon electrode (GCE), as indicated in Figure 1C. We suggest that the fabricated electrochemical sensor could exhibit at least two advantages compared to other kinds of sensors. First, the fabricated PAN–PtNPs hybrid membrane has a 3D porous structure, which could reveal higher surface area and more active sites for analytes. In addition, the nanoscale pores in the membrane could promote the adsorption and diffusion of reactants. Second, the electrostatic assembly could mediate the ordered binding of PtNPs along PAN nanofibers with relatively high density [33,34], which in some way could improve the sensitivity of the current response and enhance the sensing performance. 

## 2. Results and Discussion

### 2.1. Morphological Characterizations of PtNPs and PAN–PtNPs Nanofibers

**Figure 2:**
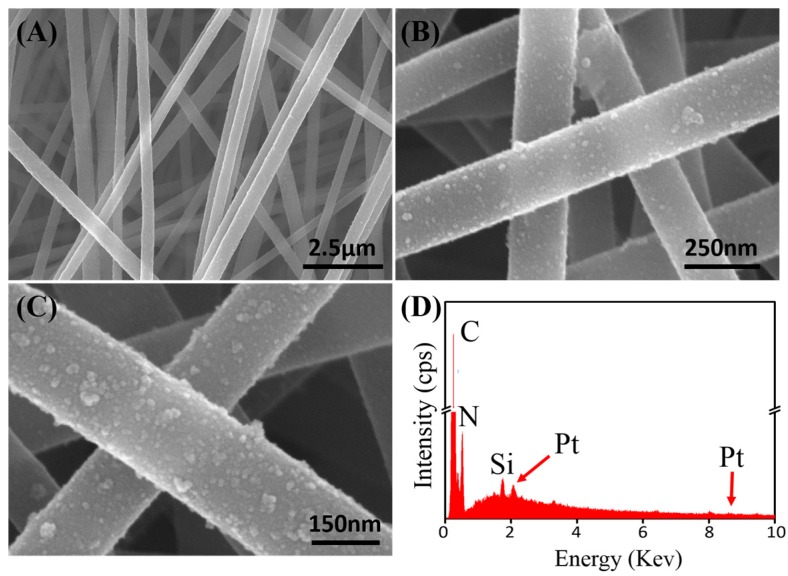
(**A**) SEM image of electrospun PAN/APS nanofibers; (**B**,**C**) SEM images of PAN–PtNPs hybrid nanofibers with different magnifications; (**D**) Corresponding energy dispersive X-ray detector (EDX) analysis.

Electrospinning is a simple and effective technique to create PMNFs. In this work, we found that the introduction of APS into the PAN solution did not affect the formation of uniform PAN nanofibers, which is proved firstly by the scanning electron microscopy (SEM) characterization. Figure 2A shows the typical SEM image of the electrospun PAN/APS hybrid nanofibers, which reveal high uniformity and linear structure with a diameter of a few 100 nm. After the electrostatic assembly of PtNPs along the fabricated PAN/APS nanofibers, it can be found that the resulted PAN–PtNPs hybrid nanofibers kept the original structure, similar to the PAN/APS nanofibers (Figure 2B), which exhibits a uniform diameter in the range of 200 to 300 nm and is decorated with many PtNPs. A further magnified SEM image (Figure 2C) indicates that the PtNPs were adsorbed uniformly on the surface of electrospun PAN/APS nanofibers due to the electrostatic self-assembly of PtNPs along PAN nanofibers. To identify the successful conjugation of PtNPs along PAN nanofibers, we utilized an energy dispersive X-ray detector (EDX) spectrum to detect the main elements of the final PAN–PtNPs hybrid nanofibers. As shown in Figure 2D, four elements, C, N, Si, and Pt, could be found in the electrospun nanofibers. In particular, two characteristic peaks of Pt (2.2 and 9.4 keV) in the EDX spectrum clearly prove the existence of PtNPs, as well as identify that the electrostatic assembly strategy is effective for creating PAN–PtNPs nanofibrous membrane [35]. In addition, further calculation indicated that the Pt element is about 5.61% of the whole amount of hybrid nanofibers, exhibiting a high density of PtNPs on the surface of PAN nanofibers. 

**Figure 3:**
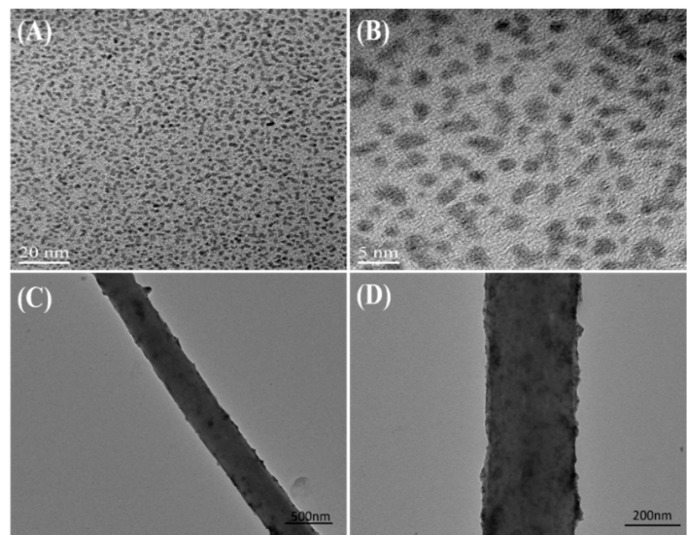
(**A**,**B**) High-resolution transmission electron microscopy (HRTEM) images of the synthesized negative-charged PtNPs; (**C**,**D**) TEM images of electrospun PAN–PtNPs with different magnifications.

Transmission electron microscopy (TEM) was further utilized to characterize the synthesized PtNPs and PAN–PtNPs hybrid nanofibers, and the obtained images are shown in Figure 3. The prepared PtNPs have a uniform size (about 2–4 nm) and there is no obvious aggregation, as shown in Figure 3A,B. We suggest that the role of glycol in the synthesis process mediated the nucleation and growth of PtNPs, and at the same time the polymer was adsorbed on the surface of PtNPs, inhibiting their aggregation. Low-concentrated PAN–PtNPs hybrid nanofibers were deposited on a Cu grid for single nanofiber analysis, and the typical TEM images are shown in Figure 3C,D. It can be found that many PtNPs were loaded onto the PAN nanofibers due to in situ precipitation as well as the electrostatic interaction between the amine groups and PtNPs. We suggest that the electrostatic assembly of PtNPs along PAN nanofibers is simple and effective for the fabrication of functional PAN–PtNPs hybrid nanofibers.

### 2.2. Property Characterizations of PAN–PtNPs Hybrid Nanofibers

**Figure 4:**
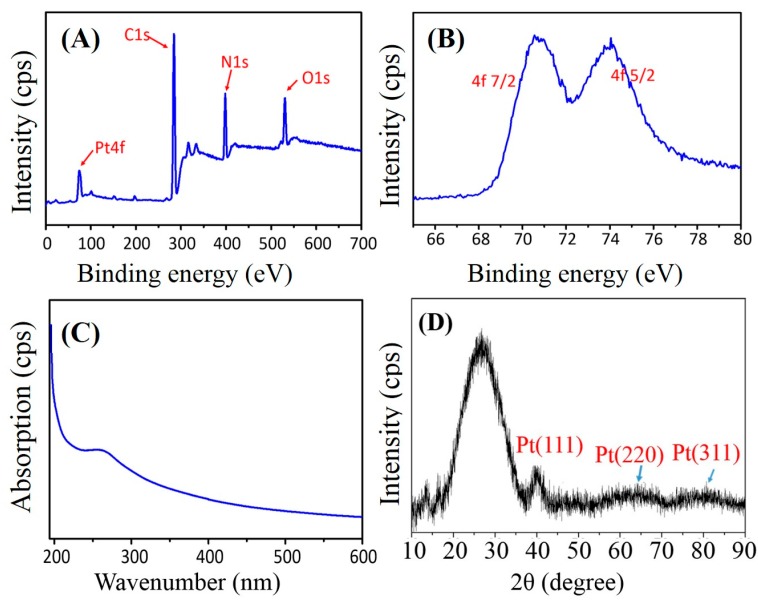
Structural characterizations of electrospun PAN–PtNPs hybrid nanofibers: (**A**,**B**) X-ray photoelectron spectrum (XPS) spectra; (**C**) UV–Vis spectrum, and (**D**) XRD pattern. Cps means counts per second.

A few structure-characterization techniques, including X-ray photoelectron spectrum (XPS), ultraviolet (UV)-visible spectrum, and Power X-ray diffraction (XRD), were utilized to identify the formation and detailed structure of PtNPs on PAN nanofibers. Figure 4A presents the large-range XPS spectrum of the electrospun PAN–PtNPs nanofiber materials, and five main elements from the sample, Pt, Si, C, O, and N, can be seen clearly. Here, we suggest that the C and N elements arise due to the use of PAN, and the Si and O elements are ascribed to the introduction of APS. To prove that the Pt element is really from the formed PtNPs, we conducted a further XPS analysis on the obtained Pt peak in Figure 4A. As indicated in Figure 4B, two characteristic peaks of PtNPs (71 and 74.3 eV) were found, which could be assigned to the typical planes (4f_7/2_ and 4f_5/2_) of pure PtNPs [36,37]. XPS characterization provided a simple and direct analysis of the synthesized metallic NPs, and here the successful synthesis of PtNPs along PAN nanofibers is identified.

The typical ultraviolet absorption peak of Pt (IV) is 264 nm (wavenumbers), and is an intense absorption peak [38]. Figure 4C shows the corresponding ultraviolet (UV)-visible spectrum of the fabricated PAN–PtNPs hybrid nanofibers. The typical characteristic peak at 261 nm is weak, which could be ascribed to the reduction of Pt (IV). To ensure the crystalline of the created PtNPs, the fabricated PAN–PtNPs nanofibrous membrane was further analyzed with power XRD, and a typical XRD pattern is given in Figure 4D. A strong peak at about 26° could be found due to the high C content in the sample. Although three other peaks at about 39.7°, 46.2°, and 67.5° are not as strong and clear as that at 26°, they still correspond to the previously identified (111), (200), and (220) planes of PtNPs. Based on the above results and analysis, it can be determined that pure PtNPs with high quality were synthesized and conjugated onto the surface of PAN nanofibers successfully.

### 2.3. Electrochemical Sensing H_2_O_2_

Although pure PMNFs have no effects on the sensing performance of the fabricated electrochemical sensors, previous studies have indicated that synthesized PtNPs could greatly increase the electrocatalytic activity of electrodes [35,36]. Therefore, the fabricated PAN–PtNPs hybrid nanofibers in this work may have potential application as a high-performance nanomaterial for creating a non-enzymatic electrochemical H_2_O_2_ sensor. To prove this, a GCE was modified by the final PAN–PtNPs nanofibrous membrane and the fabricated PAN–PtNPs/GCE was utilized for the following electrochemical tests. 

**Figure 5:**
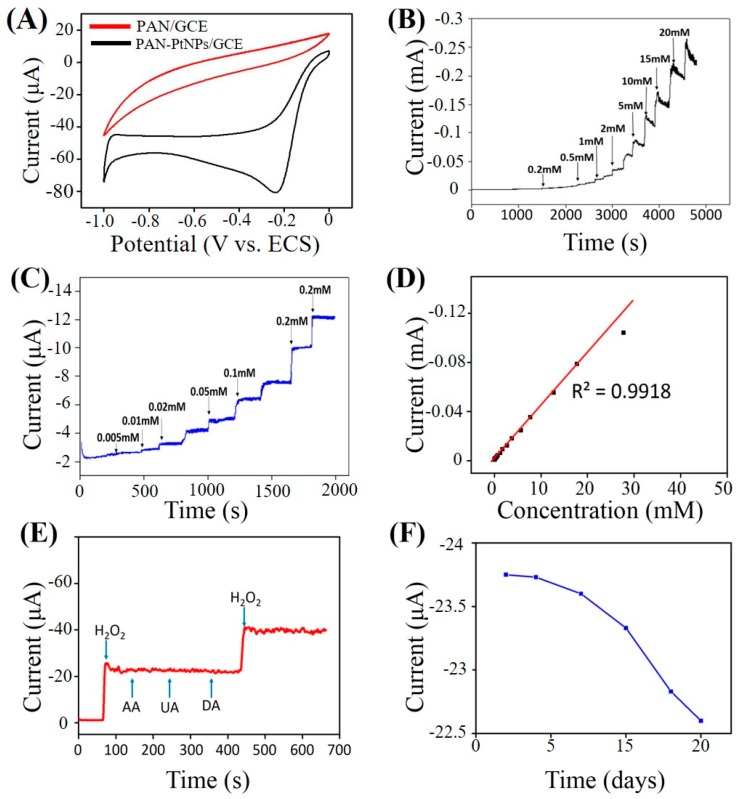
PAN–PtNPs/glassy carbon electrode (GCE) for sensing test (scan rate: 50 mV/s, PBS, PH = 7.4): (**A**) Typical cycle voltammetry (CV) curves of both PAN/GCE and PAN–PtNPs/GCE; (**B**,**C**) Current-time (I-T) response of PAN–PtNPs/GCE with different detection ranges; (**D**) Corresponding linear current-concentration relationship; (**E**) Selectivity; and (**F**) Stability.

To prove the improved electrocatalytic activity of the fabricated PAN–PtNPs/GCE compared to the pure PAN/GCE, the cycle voltammetry (CV) curves of both GCEs under the same electrochemical conditions (0.5 mM H_2_O_2_, scan rate: 50 mV/s) were measured, and the obtained CV curves are shown in Figure 5A. It is clear that the PAN/GCE shows no oxidization or reduction peaks, but the PAN–PtNPs/GCE created a significant reduction peak at about −0.24 V. We suggest that the obtained CVs confirm the importance of PtNPs in the catalytic activity of PAN–PtNPs/GCE towards H_2_O_2_.

Under an optimal applied potential of −0.24 V, the current-time (I-T) response of the fabricated PAN–PtNPs/GCE was carried out by adding H_2_O_2_ with various concentrations, and the long-term I-T curve is shown in Figure 5B. It is clear that the current response kept stable throughout the beginning 3000 s, while afterwards a light current vibration could be observed. Figure 5C presents a stable I-T response area until 2000 s by adding H_2_O_2_ with various concentrations. The corresponding calibration towards the I-T curve indicates that the fabricated electrochemical sensor has a linear detection range from 5 μM to 53 mM (Coefficient of determination (R^2^) = 0.9918). The further calculation of the detection limit of the fabricated sensor through the classic equation (detection limit = 3 σ/*S*, in which σ is the standard deviation of the current response and S is the slope of the calibrated line) indicates that a detection limit as low as 1.46 μM (signal to noise ratio (S/N) = 3) could be obtained. Compared to previous studies on the use of polymer nanofibrous materials for H_2_O_2_ sensing [28,33,39,40], our H_2_O_2_ sensor in this study exhibits a lower limit of detection (LOD), as indicated in Table 1.

A previous study has shown that a few interferences such as ascorbic acid (AA), uric acid (UA), and dopamine (DA) could affect the sensing of H_2_O_2_ by the electrochemical technique [22]. Therefore, the sensor selectivity of the PAN–PtNPs/GC-based H_2_O_2_ sensor was investigated by adding H_2_O_2_, AA, UA, and DA, and again H_2_O_2_, respectively, as indicated in Figure 5E. The corresponding current response proves that the addition of three structure-similar chemicals into the detection system did not affect the sensing of H_2_O_2_ significantly. Another parameter, long-term stability, is also crucial for determining the sensing performance of an electrochemical sensor. Therefore, the long-term stability of the fabricated PAN–PtNPs/GCE-based sensor was further tested, and the result is presented in Figure 5F. Within a detection period of 15 days, the fabricated sensor was utilized to detect 0.5 mM H_2_O_2_ for every two or three days. It is clear that the current response decreased about 5% after six time measurements. Based on these results, we suggest that the fabricated PAN–PtNPs/GCE-based sensor has acceptable selectivity and stability under the test conditions, but the practical detection of real samples by this sensor should be further investigated in the next step. 

**Figure 6:**
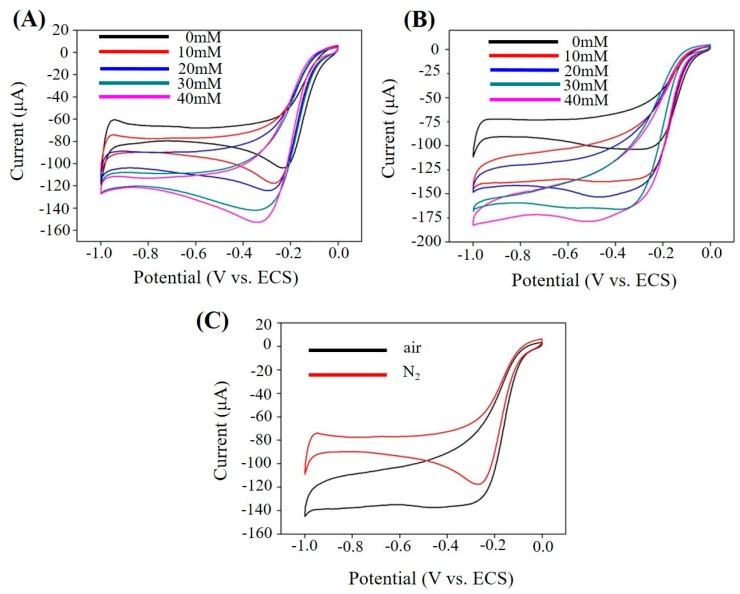
PAN–PtNPs/GCE for sensing test (scan rate: 50 mV/s, PBS, PH = 7.4): (**A**) Typical CV curves of PAN–PtNPs/GCE with different H_2_O_2_ concentrations under N_2_ atmosphere; (**B**) Typical CV curves of PAN–PtNPs/GCE with different H_2_O_2_ concentrations under air atmosphere; (**C**) Corresponding CV curves with the same H_2_O_2_ concentration under air and N_2_ atmosphere.

Figure 6 shows the contrast image of atmospheres. Figure 6A,B present the CV curves of PAN–PtNPs/GCE with different H_2_O_2_ concentrations under N_2_ atmosphere and air, it could be found that the basic tendency of the curves is similar due to the same redox reactions. However, O_2_ is a production in this reaction process, and its existence hinders the reaction, as shown in Figure 6C. The peak in air is a little bit larger than that in N_2_ atmosphere.

## 3. Materials and Methods 

*Reagents and materials.* PAN (Mw = 150,000) was provided by J&K Scientific Ltd., Beijing, China. *N*,*N*-Dimethylformamide (DMF, >99.8%), sodium hydroxide (NaOH, ≥99.0%), chloroplatinic acid hydrate (H_2_PtCl_6_·6H_2_O, ≥99.9%), and Nafion solution were purchased from Sigma-Aldrich (St. Louis, MO, USA). Disodium hydrogen phosphate (Na_2_HPO_4_), sodium dihydrogen phosphate (NaH_2_PO_4_), ethanol, ascorbic acid (AA), uric acid (UA), and dopamine (DA) were purchased from Beijing Chemicals Co., Ltd. (Beijing, China). H_2_O_2_ (analytical grade, 30% aqueous solution) was supplied by Tianjin Dong fang Chemical Plant (Tianjin, China). The water used was purified through a Millipore system (18.2 MΩ·cm).

*Electrospinning preparation of PAN/APS nanofibers.* First, 1.5 g PAN was dissolved in 15 mL DMF at 80 °C and stirred for 2 h until completely dissolved to prepare the electrospinning solutions. Then, 0.18 g APS was added into the spinning solution until PAN was cooled to room temperature, followed by further stirring for 6 h. The mass ratio of APS to PAN was adjusted to 12%. The total concentration of all the solutions was 20 wt %, which is homogeneous and stable. The preparation of PAN/APS nanofibers was performed on a home-designed electrospinning apparatus produced by Beijing Technova Technology Co., Ltd (Beijing, China). For the electrospinning, 5 mL mixed solution was loaded into a 10 mL syringe (18-gauge blunt tip needle) and an electrospinning rate of 0.1–0.3 mL/h was set. In addition, an applied voltage (10 kV) and distance (about 12 cm) were utilized. The electrospun PAN/APS hybrid nanofibrous membrane was placed under ambient conditions for 48 h and then heated in a ventilated oven at 60 °C for 24 h for the subsequent conjugation with PtNPs.

*Synthesis and binding of PtNPs.* The platinum sol-containing PtNPs was prepared by a typical aqueous reduction method and used for binding. In brief, 25 mL NaOH (0.5 M) in glycol solution was first mixed with 25 mL H_2_PtCl_6_·6H_2_O (1.9 mM) under stirring. N_2_ was used to eliminate moisture and other organic byproducts in the solution system. Then, the mixed solution was heated to 160 °C for 3 h to synthesize the polymer-protected PtNPs. Natural cooling to room temperature occurred to obtain a dark brown, unprotected colloidal solution. The conjugation of PtNPs onto the electrospun PAN/APS mats was achieved by the in situ precipitation method. Specifically, the electrospun fiber mat was cut into rounds with a diameter of 3.0 cm and then immersed into 10 mL of platinum sol. Next, HCl (1 M) was added dropwise into the system until the pH of the system reached 4.0. After that, the mat was then washed five times with deionized water and dried thoroughly.

*Preparation of PAN–PtNPs/GCE.* Before the modification, GCE was polished with 1 and 0.3 μm alumina slurry, and after that, the polished GCE was further washed with ethanol and distilled water in an ultrasonic bath for 10 s, respectively. For the immobilization of nanofibrous membrane on GCE, the GCE was fixed on the drum collector, and both the drum collector and GCE were connected to the ground, and the whole process of electrospinning lasted for 2 min. Finally, the fabricated PAN–PtNPs/GCE was dried in air and stored at 4 °C for the electrochemical detection of H_2_O_2_. 

*Characterization techniques.* SEM characterization was carried out with a JSM-6700F scanning electron microscope (JEOL, Tokyo, Japan) at 20 kV. TEM measurements were performed with a Tecnai G220 transmission electron microscope (FEI, Beijing, China) with an accelerating voltage of 200 kV. XRD (Rigaku D/max-2500VB+/PC, Shanghai, China), XPS (ThermoVG ESCALAB 250, Tokyo, Japan), and Raman spectroscopy (LabRAM HORIBA JY, Edison, NJ, USA) were used to characterize the structure of samples.

*Electrochemical tests.* All electrochemical tests were performed on a CHI 660A electrochemical workstation (Chenhua, Shanghai, China) at room temperature. A conventional three-electrode system was employed with a bare GCE, a Pt wire, and a KCl-saturated calomel electrode (SCE) as the working electrode, auxiliary electrode, and reference electrode, respectively. Phosphate buffer solutions (PBS, 0.1 M, pH = 7.4) that deoxygenated with highly pure N_2_ for 20 min were used for the electrochemical test system.

## 4. Conclusions

In summary, a functional PAN–PtNPs hybrid nanofibrous membrane was fabricated by electrospinning positively charged PAN/APS nanofibers and then electrostatically assembling negatively charged PtNPs onto the nanofibers. The electrostatic assembly technique promotes the binding of PtNPs onto PAN/APS nanofibers with high order and density, which potentially enhanced their application as a non-enzymatic electrochemical H_2_O_2_ sensor. The electrochemical data indicates that the PAN–PtNPs/GCE-based sensor shows good electrocatalytic activity toward H_2_O_2_. The fabricated electrochemical H_2_O_2_ sensor exhibits a wide linear range (5 μM–53 mM), low detection limitation (1.46 μM), as well as high selectivity and stability. It is expected that the methodologies utilized in this work will promote the design and fabrication of functional nanofiber-based biomaterials and nanodevices, as well as extend their potential applications in energy storage, cytology, and biomedical engineering.

## Figures and Tables

**Table 1:** Comparison of the performance of the present work with other electrochemical H_2_O_2_ sensors.

Materials	Linear Range	Detection Limit	Reference
PU-AgNPs	0.5–30 mM	18.6 μM	[28]
PVA-AgNPs	5 μM–0.6 mM	5 μM	[38]
PAN–PtNPs	0.1–30 mM	1.9 μM	[39]
Paraffin-Silica-AuNPs	5 μM–1 mM	2 μM	[33]
PAN–PtNPs	5 μM–53 mM	1.46 μM	This work
